# Exploratory Mediation-Informed Analysis and Dose–Response Analysis of Uric Acid Reduction and Kidney Outcomes in SGLT2 Inhibitor Therapy Compared to Allopurinol

**DOI:** 10.3390/medsci14040421

**Published:** 2026-07-23

**Authors:** Tamás Jámbor, Szabolcs Péter Tallósy, Tamás Lantos, Andrea Szabó, Roland Fejes

**Affiliations:** 1 Institute of Surgical Research, Albert Szent-Györgyi Medical School, University of Szeged, 6720 Szeged, Hungary; jambor.tamas@med.u-szeged.hu (T.J.); tallosy.szabolcs@med.u-szeged.hu (S.P.T.); szabo.andrea.exp@med.u-szeged.hu (A.S.); 2 Department of Internal Medicine, Hódmezővásárhely-Makó Healthcare Center, 6900 Makó, Hungary; 3 Department of Medical Physics and Informatics, Albert Szent-Györgyi Medical School, University of Szeged, 6725 Szeged, Hungary; lantos.tamas@med.u-szeged.hu

**Keywords:** SGLT2 inhibitors, allopurinol, hyperuricemia, chronic kidney disease, estimated glomerular filtration rate, mediation analysis, retrospective analysis

## Abstract

**Background**: Hyperuricemia has long been linked to the progression of chronic kidney disease (CKD), although the role of serum uric acid (sUA) reduction in kidney preservation remains uncertain. Sodium-glucose cotransporter 2 (SGLT2) inhibitors lower sUA levels and exert renoprotective effects, but it is unclear whether these findings are mechanistically related. **Methods**: Adult patients with type 2 diabetes mellitus and hyperuricemia were included from a retrospective cohort. Patients treated with empagliflozin (*n* = 70) or dapagliflozin (*n* = 78) were pooled into a single group and compared with an allopurinol-treated group (*n* = 66). Exploratory mediation-informed analysis models assessed associations between treatment, changes in sUA (ΔsUA), and changes in estimated glomerular filtration rate (ΔeGFR) at 12 and 36 months. Dose–response and nonlinear associations between ΔsUA and ΔeGFR were additionally examined. **Results**: At 12 months, SGLT2 inhibitor treatment was associated with greater ΔsUA compared with allopurinol (*B* = 22.62, *p* = 0.040); its direct association with ΔeGFR remained significant after adjustment for ΔsUA (*B* = −6.14, *p* < 0.001). At 36 months, the association of treatment with ΔsUA was no longer significant, whereas association with ΔeGFR persisted (*B* = −11.8, *p* < 0.001). No dose–response or nonlinear relationship was identified between ΔsUA and ΔeGFR, while treatment remained an independent predictor of ΔeGFR at both time points. **Conclusions**: Reductions in sUA do not appear to mediate the renoprotective effects of SGLT2 inhibitors. The preservation of kidney function associated with SGLT2 inhibitor therapy is likely driven by mechanisms independent of sUA lowering.

## 1. Introduction

Hyperuricemia (HUA), the abnormal elevation of serum uric acid (sUA) levels (>416 µmol/L in men and >357 µmol/L in women), affects more than 800 million people worldwide [1,2]. Due to the presumed pathogenic effects of HUA’s underlying cardiovascular (CV) and kidney pathologies, antihyperuricemic agents have substantial clinical relevance and economic impact [3]. Nevertheless, major clinical trials found no significant association between routine sUA-lowering therapy and favorable CV and kidney outcomes [4,5,6]; consequently, HUA may represent an epiphenomenon or a marker of clinical risk [7]. Regarding the uncertainty surrounding the etiology of HUA, it is worth highlighting the latest KDIGO guidelines on the management of chronic kidney disease (CKD), which do not recommend initiating antihyperuricemic treatment in asymptomatic HUA for slowing CKD progression [8].

Sodium-glucose cotransporter 2 (SGLT2) inhibitors, such as dapagliflozin or empagliflozin, are antidiabetic, cardioprotective, and renoprotective agents [9]. Their uricosuric effect has been shown to reduce sUA, as reported by our group and others [10]. Based on our data, it was also established that, compared with allopurinol―a standard antihyperuricemic agent―SGLT2 inhibitors not only achieved a comparable sUA reduction, but also resulted in a significant preservation of estimated glomerular filtration rate (eGFR) and a more favorable clinical outcome profile. Moreover, the effect of these agents proved robust across the entire study population; no modification of therapeutic efficacy based on baseline parameters was observed, and while baseline sUA was validated as a predictor of the outcomes, its role as a mediator remains uncertain [11]. Although a reduction in sUA induced by SGLT2 inhibitors has been proposed as a potential contributor to their renoprotective effects, it remains unclear whether this represents a causal pathway or reflects a correlated treatment response.

Based on a reanalysis of our previously established dataset from a different perspective, the primary objective of this study was to determine whether the reduction in sUA observed during SGLT2 inhibitor treatment mediates the treatment-associated renoprotective effect, compared with allopurinol. To this end, we performed exploratory mediation-informed analysis using mediation-based statistical models at two predefined time points: the 12-month analysis was predefined as the primary analysis to capture early treatment effects, while the 36-month analysis was conducted as a secondary, longer-term evaluation. As a secondary objective, we examined whether there was a dose–response relationship between the degree of sUA reduction and changes in eGFR, independent of treatment, while taking baseline clinical characteristics into account.

## 2. Materials and Methods

### 2.1. Study Population and Data Sources

The current study is a secondary analysis of a previously assembled retrospective cohort, using the original dataset described in earlier publications [10]. The cohort included Caucasian patients aged ≥18 years treated at an outpatient diabetes clinic in Hungary. Individuals were included who were initiated on either an SGLT2 inhibitor (empagliflozin 10 mg/day or dapagliflozin 10 mg/day) or allopurinol (100 mg/day) between 1 January 2017 and 1 January 2020. Patients who underwent dose escalation in any treatment group during follow-up were excluded to ensure a stable exposure regimen throughout the observation period. Detailed information on patient selection, including inclusion/exclusion criteria and final sample sizes, is provided in Supplementary Material S1 and Table S1. HUA was defined as sUA > 416 µmol/L in men and >357 µmol/L in women [2].

Patients were excluded if they had baseline sUA below the diagnostic threshold, received concurrent use of allopurinol and SGLT2 inhibitors, had treatment dose escalation during follow-up, or had incomplete records.

The study was conducted in accordance with the principles of the Declaration of Helsinki and its later amendments. Ethical approval was obtained from the Institutional Review Board of the Hódmezővásárhely-Makó Healthcare Center and the Hungarian National Public Health Center Institutional Committee of Science and Research Ethics (NNGYK/65897-2/2025; approved 28 October 2025). All data were anonymized prior to analysis, and the requirement for informed consent was waived due to the retrospective nature of the study.

### 2.2. Statistical Analysis

#### 2.2.1. Descriptive Analysis, Software

Normality was assessed with the Shapiro–Wilk test. Normally distributed variables are presented as mean ± SD, while skewed non-normal variables are displayed as median (25th–75th percentile). Categorical variables are summarized as *n* (%), with between-group comparisons by Fisher’s exact test. All statistical tests were two-sided, and a *p* value <0.05 was considered statistically significant. Effect estimates are presented with 95% confidence intervals (CIs). Analyses were performed using GraphPad Prism (version 8.0.1; GraphPad Software, Boston, MA, USA) and JASP software (v.0.95.4, University of Amsterdam, Amsterdam, The Netherlands).

#### 2.2.2. Exposure, Mediator, Outcome Definitions, and Exploratory Mediation-Informed Analysis

For the primary pathway-oriented mediation framework analyses, treatment was modeled as a binary variable. Data from patients treated with empagliflozin or dapagliflozin were pooled and compared with those from patients treated with allopurinol alone. The mediator was defined as the absolute change in sUA. The primary outcome was the change from baseline (Δ) in eGFR over time. A continuous measure was used to avoid information loss. Results were adjusted forage, sex, BMI, duration of diabetes, baseline HbA1c, baseline sUA, and baseline eGFR. Additional variables included hypertension, CKD, coronary artery disease, and insulin therapy in sensitivity analyses. Treatment was coded as a binary variable, with T = 1 for patients receiving SGLT2 inhibitors and T = 0 for patients receiving allopurinol monotherapy. In theory, the reduction in sUA levels due to treatment with SGLT2 inhibitors and the preservation of eGFR may be interrelated; the treatment-induced ΔsUA could contribute to the observed ΔeGFR, representing a mediated indirect effect. However, a direct renoprotective effect of SGLT2 inhibitors, independent of ΔsUA, cannot be excluded. To quantify the proportion of the total treatment effect that is mediated through ΔsUA, a mediation model can be applied, allowing for the estimation of both direct and indirect effect components based on the following regression-based approaches:

$$M=\;a_{0}+\;a_{1}T+\;a_{2}C+\;\varepsilon_{1}$$

where *M* represents the mediator variable (ΔsUA), T the treatment, *C* the vector consisting of the covariates, *a*_0_ the intercept, *a*_1_ the regression coefficient describing the association between treatment allocation and ΔsUA, *a*_2_ the vector comprising the coefficients of the included covariates, and *ε*_1_ the residual error term representing unexplained variability.

$$Y=b_{0}+c^{\prime }T+b_{1}M+b_{2}C+\varepsilon_{2}$$

where *Y* represents the outcome variable (ΔeGFR), T the treatment, *M* the mediator variable (ΔsUA), *C* the vector consisting of the covariates, $b_{0}$ the intercept, *c*′ the regression coefficient representing the direct association between treatment allocation and ΔeGFR independent of the mediator, *b*_1_ the regression coefficient describing the association between the mediator and ΔeGFR after adjustment for treatment and covariates, *b*_2_ the vector comprising the coefficients of the included covariates, and *ε*_2_ the residual error term representing unexplained variability. It should be noted that this analytical approach does not provide complete certainty, as unmeasured confounders and the retrospective nature of the data collection may influence the validity of the estimated effects.

The mediator model regressed ΔsUA on treatment and covariates, while the outcome model regressed ΔeGFR on treatment, ΔsUA, and the same covariates. Indirect, direct, and total effects and mediated proportion (i.e., indirect effect divided by the total effect) were calculated. In addition, 95% confidence intervals (95% CIs) for all parameters were obtained by nonparametric bootstrapping with 5000 replications.

#### 2.2.3. Dose–Response Analysis

The relationship between sUA reduction and ΔeGFR was assessed using multiple linear regression. We tested for interaction between sUA reduction and treatment group. If the interaction term was not significant, a common slope was assumed. Potential nonlinearity was assessed by adding a quadratic term for sUA reduction to the adjusted linear regression model. Nonlinearity was considered present if the quadratic term was statistically significant. In exploratory analyses, sUA reduction was divided into quantiles.

## 3. Results

### 3.1. Exploratory Mediation-Informed Analysis

**Figure 1:**
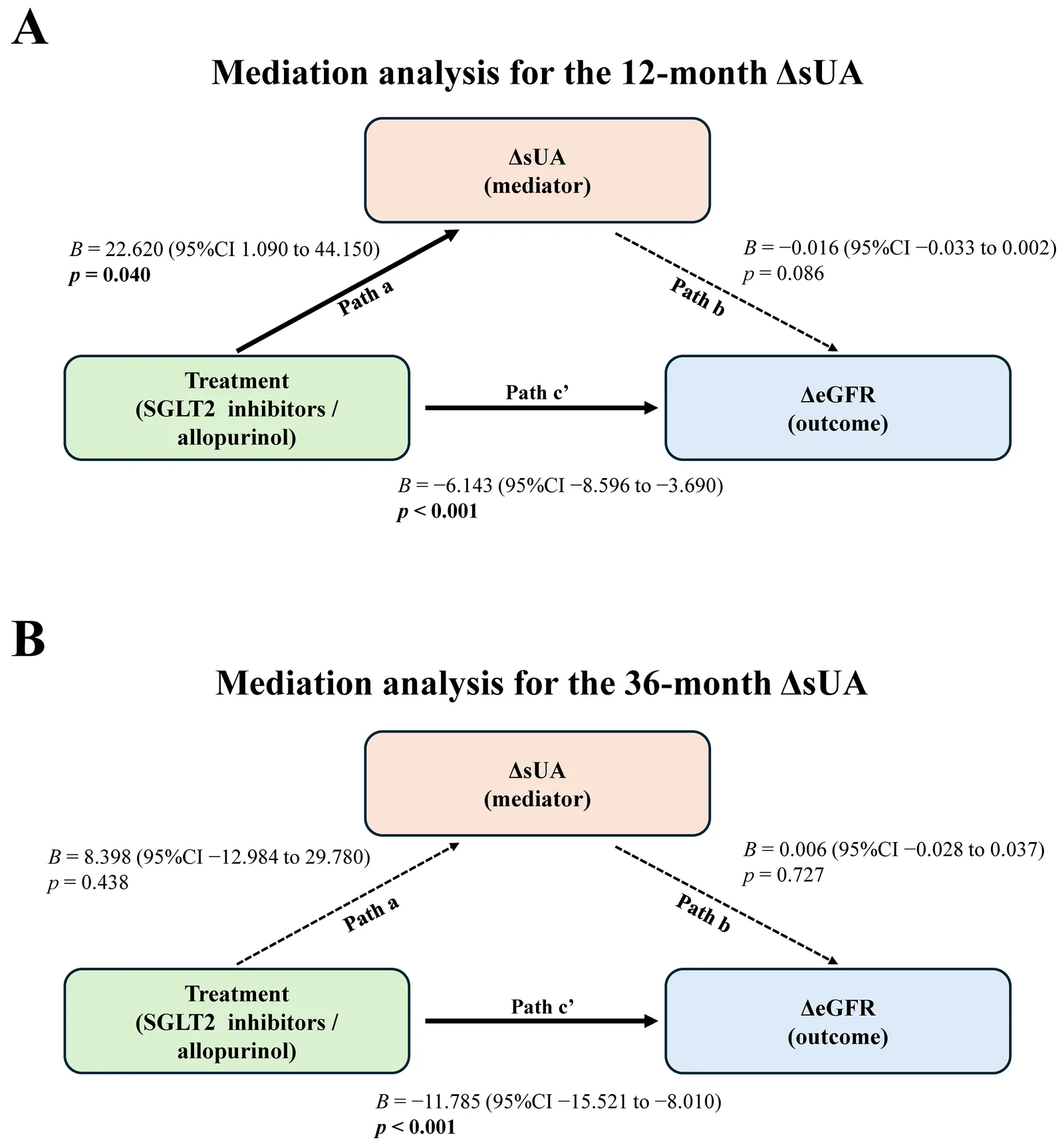
Exploratory pathway-mediated analysis of the association between treatment (SGLT2 inhibitors versus allopurinol) and ΔeGFR as the outcome, with ΔsUA as the mediator, at 12 months (**A**) and 36 months (**B**). Path a = effect of treatment on the mediator; Path b = effect of the mediator on the outcome; Path c’ = direct effect of treatment on. Unstandardized coefficients (*B*) with 95% confidence intervals and *p*-values are shown. Solid arrows indicate statistically significant associations (*p* < 0.05); dashed arrows indicate non-significant associations.

Twelve-month analysis: A total of 165 patients were included in the analysis (SGLT2 inhibitor group *n* = 102, allopurinol group *n* = 63) after clinically relevant attrition. Detailed data on attrition during follow-up, including treatment discontinuation and death, are available in the original publication [10]. The overall model was statistically significant (*F*(8, 156) = 4.695, *p* < 0.001) and explained 19.4% of the variance in the outcome (*R*^2^ = 0.194, adjusted *R*^2^ = 0.153, *p* < 0.001). Treatment allocation showed a significantly positive association with a greater ΔsUA (*B* = 22.620, *SE* = 10.900, *β* = 0.161, *t* = 2.075, *p* = 0.040, 95% CI 1.090 to 44.150). Bootstrap analysis with 5000 replications (bias-corrected and accelerated; BCa) confirmed the positive direction of the treatment effect on ΔsUA (*B* = 23.405, bootstrap *SE* = 11.948, 95% BCa CI −3.732 to 43.960, *p* = 0.092). However, the association did not reach statistical significance in the bootstrap analysis, whereas baseline sUA remained a strong and highly significant negative predictor (*B* = −0.426, bootstrap *SE* = 0.099, 95% BCa CI −0.657 to −0.262, *p* < 0.001). In the outcome model, the direct effect of treatment on ΔeGFR remained significant after adjustment for ΔsUA (*B* = −6.14, 95% CI −8.60 to −3.69, *p* < 0.001), whereas the association between ΔsUA and ΔeGFR was not statistically significant (*B* = −0.016, 95% CI −0.033 to 0.002, *p* = 0.086). The outcome model was statistically significant (*F*(9, 155) = 4.885, *p* < 0.001) and accounted for 21.4% of the variance (*R*^2^ = 0.214, adjusted *R*^2^ = 0.168). The mediated proportion in the whole model was 5.56%. Figure 1A illustrates the mediation analysis of 12-month data.

Thirty-six-month analysis: A total of 129 patients were included in the analysis (SGLT2 inhibitor group *n* = 78, allopurinol group *n* = 51) after clinically relevant attrition. Detailed data on attrition during follow-up, including treatment discontinuation and death, are available in the original publication [10]. The overall model was statistically significant (*F*(8, 120) = 5.379, *p* < 0.001) and explained 26.4% of the variance in the outcome (*R*^2^ = 0.264, adjusted *R*^2^ = 0.215, *p* < 0.001). Treatment was no longer significantly associated with ΔsUA (*B* = 8.398, *SE* = 10.799, *β* = 0.066, *p*= 0.438, 95% CI −12.984 to 29.780). Baseline sUA remained a strong negative predictor (*B* = −0.473, *p* < 0.001). Bootstrap analysis confirmed these findings (*p* = 0.349). In the outcome model, similarly, no association was observed between ΔsUA and ΔeGFR (*B* = 0.006, 95%CI−0.026 to 0.037, *p* = 0.727). In contrast, the direct effect of treatment on ΔeGFR remained strong and statistically significant (*B* = −11.785, 95%CI−15.521 to −8.010, *p* < 0.001). The model significantly predicted ΔeGFR (*F*(9, 119) = 7.144, *p* < 0.001) and accounted for 35.1% of the variance (*R*^2^ = 0.351, adjusted *R*^2^ = 0.302). The mediated proportion in the whole model was −0.57%.

These findings did not provide evidence of a mediation effect and suggest that the sUA-lowering effect does not contribute to long-term kidney outcomes. Exact indirect, direct, and total effects, along with mediated proportions with 95% CIs, are presented in Table 1. Figure 1A,B illustrate the mediation analysis of 12-month and 36-month data, respectively.

### 3.2. Dose–Response Analysis

At 12 months, no significant association was observed between the magnitude of sUA reduction and ΔeGFR (unstandardized coefficient: *B* = −0.015, 95% CI −0.042 to 0.011, *p* = 0.253). In contrast, treatment remained a strong independent predictor of ΔeGFR at 12 months (*B* = −6.15, 95% CI −8.63 to −3.67, *p* < 0.001). Furthermore, at 36 months, no significant association was observed between the magnitude of sUA reduction and ΔeGFR (*B* = 0.003, 95% CI −0.037 to 0.044, *p* = 0.878). In contrast, treatment remained a strong independent predictor of ΔeGFR at 36 months (*B* = −11.73, 95% CI −15.52 to −7.95, *p* < 0.001). At 12 months, the quadratic ΔsUA term was not statistically significant (*B* = 4.02 × 10^−5^, *p* = 0.978). Similarly, at 36 months, no evidence of nonlinearity was observed (*B* = −4.11 × 10^−5^, *p*= 0.849).

## 4. Discussion

Both HUA and CKD are increasingly prevalent conditions that often coexist. Given this co-occurrence and their seemingly overlapping pathophysiological features, HUA has long been hypothesized as a potential driver of CKD onset and progression [11]. Allopurinol, a well-known xanthine oxidase inhibitor, lowers sUA by reducing urate production. Nevertheless, large-scale randomized controlled trials have failed to validate this causality: the PERL and CKD-FIX trials evaluating allopurinol, as well as the FEATHER trial with febuxostat, consistently failed to show a significant impact on the rate of kidney function decline, notwithstanding the successful reduction in sUA levels [4,5,6]. While these findings do not exclude a contributory role of HUA in CKD pathogenesis, the indication for routine antihyperuricemic treatment for this purpose is questionable. This cautious perspective is reflected in current KDIGO guidelines, which refrain from recommending antihyperuricemic treatment solely to mitigate CKD progression in cases of asymptomatic HUA [8]. Importantly, CKD itself represents a high CV risk, which remains a primary determinant of pharmacological management in this patient population [12,13].

SGLT2 inhibitors have emerged as a cornerstone of CKD management, primarily by modulating glomerular microhemodynamics to reduce intraglomerular pressure [14]. In addition, they also exert clinically relevant metabolic effects, including a reduction in sUA [15,16,17]. It is worth emphasizing that this metabolic effect cannot be considered independent of hemodynamic changes but is closely intertwined with them. For instance, glycosuria-induced increases in tubular glucose concentration modify GLUT9 function, thereby inhibiting uric acid reabsorption [18]. In addition, enhanced natriuresis and altered organic anion excretion influence URAT1 activity [19,20], while increased tubular flow reduces the contact time available for reabsorption [21]. Taken together, the antihyperuricemic effect of SGLT2 inhibitors reflects the interplay between tubular transport processes and kidney hemodynamics, which fits well with the pleiotropic effect profile of this class of drugs [22].

Numerous clinical trials have shown that SGLT2 inhibitors achieve clinically meaningful reductions in sUA, consistent with findings from our group [10,11]. In our cohort, both empagliflozin and dapagliflozin achieved reductions in sUA comparable to those observed with allopurinol 100 mg once daily. In contrast, eGFR trajectories differed between treatment groups: a decline was observed in the allopurinol-treated group, whereas eGFR remained stable in patients receiving SGLT2 inhibitors. Detailed longitudinal eGFR results (group-level and patient-level), including the 12- and 36-month assessments, are reported in the primary publication [10]. In additional analyses, including stratification by phenotype, no baseline parameter was identified that influenced this difference. Comparing our results with available literature data, the hypothesis reemerges that sUA alone does not play a causal, mechanistic role in the decline in eGFR [4,5,6,23,24]. This interpretation is supported by the observation that two interventions producing similar reductions in sUA may have divergent kidney outcomes: an agent with established renoprotective effects slows CKD progression [25,26], whereas one without such effects does not, and may even be associated with a decline in kidney function [4,5,6].This apparent dissociation highlights a fundamental mechanistic inconsistency with potential clinical relevance. Indeed, if antihyperuricemic treatment does not provide a meaningful renoprotective effect, the question arises as to whether its routine use is justified solely for the purpose of slowing CKD progression. This consideration is particularly relevant given that allopurinol use may be associated with increased risk in certain ranges of kidney function [27].

In the present study, we examined whether ΔsUA was associated with the renoprotective effect of SGLT2 inhibitor treatment using a mediation-informed pathway framework. To investigate this, we performed sequential multivariable regression models within a mediation-informed analytical framework. Based on the literature indicating no meaningful differences between empagliflozin and dapagliflozin, and on our previous findings showing no relevant difference in clinical effectiveness, the two agents were analyzed as a single group to increase statistical power. Although SGLT2 inhibitor treatment resulted in a significant reduction in sUA levels over 12 months, this was not associated with a meaningful improvement in kidney function. In other words, a relationship between treatment and the reduction in sUA was detectable; however, ΔsUA no longer showed a significant correlation with ΔeGFR. Accordingly, the model-estimated indirect effect mediated through sUA was negligible. The 36-month analysis further reinforced this observation; by this point, the association between treatment and the reduction in uric acid levels was no longer significant, while the estimated indirect effect continued to weaken.

Our results showed no dose–response relationship between the degree of sUA reduction and ΔeGFR. Although a weak, statistically nonsignificant trend was observed in the 12-month data, it did not reach statistical significance and did not persist over time. By 36 months, the association was no longer detectable, and the estimated effect approached practically zero. Furthermore, formal testing using quadratic regression terms did not identify evidence of nonlinear, threshold-dependent, or cumulative renoprotective associations related to sUA reduction. These analyses did not identify evidence of a threshold-dependent or cumulative renoprotective association related to sUA reduction. It is particularly important to note that even at 12 months―when the antihyperuricemic effect of SGLT2 inhibitors is most pronounced―no statistically meaningful indirect pathway was detectable, while at 36 months, neither ΔsUA nor its mediating role was interpretable.

These results strongly suggest that the renoprotective effect of SGLT2 inhibitors is unlikely to be primarily explained by a reduction in sUA. In contrast, the estimated direct effect of treatment on eGFR persisted over time and even became more pronounced, suggesting that the underlying renoprotective mechanisms are independent of ΔsUA. These mechanisms are well established and have been extensively described in the literature. The absence of a dose–response relationship is particularly informative. If sUA reduction played a significant role in preserving kidney function, a greater reduction in sUA would be expected to translate into a more favorable eGFR trajectory. Since no such association was detectable at either early or later time points, our data suggest that ΔsUA may represent a concomitant metabolic biomarker of SGLT2 inhibitor treatment rather than a relevant indirect pathway of the renoprotective effect.

Theoretically, concomitant treatment with an SGLT2 inhibitor and allopurinol could provide complementary effects when both cardiorenal protection and urate-lowering therapy are clinically indicated. The combination may improve urate control through distinct pathways, while the expected kidney benefit would primarily derive from SGLT2 inhibition. A recent small retrospective study in patients with gout found that combined SGLT2 inhibitor and urate-lowering therapy (predominantly allopurinol) was associated with an additional median serum urate reduction of 0.85 mg/dL (≈51 µmol/L) and high rates of urate-target achievement. However, this study was not designed to establish improved kidney outcomes, and robust evidence for additive or synergistic renoprotection from the combination remains unavailable [28].

Other urate-lowering agents differ in both mechanism and supporting clinical evidence. Febuxostat is a non-purine selective xanthine oxidase inhibitor that reduces uric acid production, similarly to allopurinol. Although potential CV and kidney benefits have been proposed, CV outcome data remain inconsistent. CARES reported higher CV and all-cause mortality with febuxostat than with allopurinol despite similar rates of the primary composite CV endpoint [29], whereas FAST demonstrated cardiovascular non-inferiority without an excess mortality risk [30]. The FREED trial suggested a favorable effect on a broader composite CV and kidney outcome, but these findings do not establish a direct vascular protective effect [31].

Dotinurad is a selective urate reabsorption inhibitor that reduces sUA by blocking URAT1-mediated proximal tubular urate reabsorption. While clinical studies support its urate-lowering efficacy, evidence concerning vascular or kidney protection is currently based mainly on smaller studies and surrogate outcomes [32]. Robust evidence demonstrating reductions in hard cardiovascular or kidney endpoints is not yet available. Accordingly, potential vascular protective effects of febuxostat or dotinurad should be regarded as possible but unconfirmed. Neither agent was included in the present cohort, precluding any direct comparison with allopurinol or SGLT2 inhibitors. From a clinical perspective, our findings do not support the use of urate lowering alone as a reliable strategy for slowing kidney function decline. Although antihyperuricemic treatments, such as allopurinol, can reduce sUA levels, this is not consistently associated with a renoprotective effect. In contrast, SGLT2 inhibitors, through their multimodal effects that target multiple pathways, have been shown to favorably influence the progression of kidney function, which appears to be independent of ΔsUA. This clinical benefit may be particularly relevant in patients with peripheral artery disease (PAD), who represent a high-risk population with substantial CV and kidney morbidity. A recent meta-analysis of randomized controlled trial data showed that SGLT2 inhibitor therapy was associated with lower risks of CV and adverse kidney outcomes in patients with PAD, without a significant increase in the risk of amputation [33]. Our observations are consistent with current recommendations, which do not recommend the routine use of urate-lowering therapy solely for the purpose of slowing CKD progression in cases of asymptomatic HUA. Our results further reinforce this approach and suggest that, when making therapeutic decisions, it is advisable to prioritize treatments that have been proven to improve clinical outcomes.

Our study has several limitations. The study included only adult patients with T2DM from a relatively homogeneous Caucasian population, which may limit the generalizability of the results. Due to the observational nature of the study, causal inferences can only be drawn to a limited extent, and the possibility of residual confounding cannot be ruled out. Allopurinol and empagliflozin were administered at a fixed dose of 100 mg/day and 10 mg/day, respectively, without titration, precluding an analysis of dose adjustment or dose–response relationships according to baseline kidney function. Although baseline eGFR was included as a continuous covariate, potential heterogeneity related to renal-function-guided dose individualization cannot be excluded. This type of mediation analysis examines statistical associations but does not, in itself, prove a causal mechanism. Δ models are associated with inherent variability, which may weaken the detected associations, particularly in the case of smaller mediated effects. ΔsUA and ΔeGFR were measured over identical time windows; therefore, the analyses could not determine whether ΔsUA and ΔeGFR were sequential or reflected parallel treatment-related processes, and the observed associations cannot distinguish mediation from parallel treatment-related changes and should be interpreted as exploratory and hypothesis-generating rather than causal. Furthermore, although both empagliflozin and dapagliflozin belong to the same therapeutic class and share major cardiorenal mechanisms, agent-specific heterogeneity cannot be excluded in the pooled dataset. As no drug-specific sensitivity analysis was performed and the study was not powered for between-agent comparisons, the findings should be interpreted as class-level associations rather than evidence of equivalence between the two SGLT2 inhibitors. Allopurinol was administered at a fixed dose of 100 mg/day without titration. Furthermore, the lack of urine albumin-to-creatinine ratios precluded the application of the full KDIGO risk stratification (G–A staging). Consequently, the study may not have captured treatment-associated kidney effects occurring primarily through changes in albuminuria, and the kidney findings should be interpreted as reflecting eGFR-based kidney function rather than the full spectrum of kidney disease progression.

## 5. Conclusions

In conclusion, SGLT2 inhibitor therapy was associated with reductions in sUA and with more favorable eGFR trajectories in this cohort. However, changes in sUA did not statistically account for the association between treatment and eGFR preservation in the exploratory mediation-informed analyses. Consistently, no clear dose–response or nonlinear association was identified between ΔsUA and ΔeGFR. These findings suggest that the observed association between SGLT2 inhibitor therapy and eGFR preservation was not statistically explained by the magnitude of sUA reduction. Nevertheless, given the observational design, the contemporaneous assessment of changes, and the possibility of residual confounding, the results do not exclude the contribution of urate-related biological pathways and should not be interpreted as evidence of definitive mechanistic independence.

## Figures and Tables

**Table 1:** Exploratory pathway-mediated analysis of the effect of treatment on ΔeGFR through ΔsUA at 12 and 36 months. Shown are unstandardized regression coefficients (*B*), standard errors (*SE*), test statistics (*t*), *p*-values, and 95% confidence intervals for path *a*, path *b*, direct effect (*c*′), indirect effect (*a* × *b*), and total effect (*c*).

**12-Month Analysis**					
**Variable**	***B***	***SE***	***t***	***p***	**95% CI**
Path *a* (Treatment → ΔsUA)	22.620	10.900	2.075	0.040	1.090 to 44.150
Path *b* (ΔsUA → ΔeGFR)	−0.016	0.009	−1.726	0.086	−0.033 to 0.002
Direct effect (*c*′)	−6.143	1.242	−4.946	<0.001	−8.596 to −3.690
Indirect effect (*a* × *b*)	−0.362	—	—	—	—
Total effect (*c*)	−6.505	—	—	—	—
**36-Month Analysis**					
**Variable**	***B***	***SE***	***t***	***p***	**95% CI**
Path a (Treatment → ΔsUA)	8.398	10.799	0.778	0.438	−12.984 to 29.780
Path b (ΔsUA → ΔeGFR)	0.006	0.016	0.349	0.727	−0.026 to 0.037
Direct effect (*c*′)	−11.785	1.897	−6.213	<0.001	−15.521 to −8.010
Indirect effect (*a* × *b*)	0.067	—	—	—	—
Total effect (*c*)	−11.718	—	—	—	—

## Data Availability

The original contributions presented in this study are included in the article/Supplementary Materials. Further inquiries can be directed to the corresponding author.

## Supplementary Materials

The following supporting information can be downloaded at: https://www.mdpi.com/article/10.3390/medsci14040421/s1, Table S1: Clinicopathologic characteristics of patients enrolled in the study. Material S1: Schematic flowchart showing the exclusion criteria used for patient selection.
