# Supplementary material for: Exploratory Mediation-Informed Analysis and Dose–Response Analysis of Uric Acid Reduction and Kidney Outcomes in SGLT2 Inhibitor Therapy Compared to Allopurinol
Source: Med Sci (Basel). 2026 Jul 23;14(4):421. doi: 10.3390/medsci14040421 (PMC13515684; doi:10.3390/medsci14040421)

SUPPLEMENTARY MATERIAL

Mechanistic Pathway and Dose–Response Analysis of Uric Acid Reduction and Kidney Outcomes in SGLT2 Inhibitor Therapy Compared to Allopurinol

Tamás Jámbor[1,2], Szabolcs Péter Tallósy[1], Tamás Lantos[3], Andrea Szabó[1] and Roland Fejes[1,2,*]

[1] Institute of Surgical Research, Albert Szent-Györgyi Medical School, University of Szeged, Szeged 6720, Hungary

[2] Department of Internal Medicine, Hódmezővásárhely-Makó Healthcare Center, Makó 6900, Hungary

[3] Department of Medical Physics and Informatics, Albert Szent-Györgyi Medical School, University of Szeged, Szeged 6725, Hungary

***Correspondence:** Roland Fejes MD,PhD

Postal: H-6720 Szőkefalvi-Nagy Béla Street 6, Szeged, Hungary

Phone: +36 62 545 103

Supplementary Table S1. Clinicopathologic characteristics of patients enrolled in the study. Data are presented as medians with 25th–75th percentiles. *P* values of <0.05 were considered to indicate statistical significance. **p* < 0.05 vs. dapagliflozin

	Dapagliflozin (n = 78)	Empagliflozin (n = 70)	Allopurinol (n = 66)	*p* value
**Age (years)**	63.0 (54.8; 68.3)	61.5 (54.0; 68.3)	64.0 (57.0; 68.3)	0.717
**Diabetes duration (years)**	10.0 (4.0; 14.3)	10.0 (6.0; 15.0)	10.5 (4.8; 18.3)	0.608
**Body weight (kg)**	101.0 (85.8; 115.0)	96.0 (83.0; 114.3)	104.0 (89.8; 113.5)	0.218
**Body mass index (kg/m^2)**	30.5 (28.0; 35.5)	31.8 (27.5; 36.1)	32.7 (30.1; 36.8)	0.053
**Fasting plasma glucose (mmol/L)**	9.3 (7.2; 11.2)	9.3 (7.9; 12.2)	8.9 (8.5; 9.5)	0.722
**HbA_{1c} (%)**	**8.5 (7.9; 9.0)**	8.2 (7.6; 8.7)	**7.8 (7.5; 8.1)***	***<0.001**
**Total cholesterol (mmol/L)**	5.2 (4.4; 6.0)	5.5 (4.4; 6.1)	5.8 (4.6; 6.3)	0.188
**HDL-cholesterol (mmol/L)**	1.3 (1.1; 1.6)	1.3 (1.1; 1.7)	1.4 (1.1; 1.6)	0.495
**LDL-cholesterol (mmol/L)**	3.4 (2.6; 3.9)	3.4 (2.7; 4.0)	4.0 (2.8; 4.5)	0.061
**Serum uric acid (μmol/L)**	450 (418; 501)	452 (401; 497)	438 (388; 510)	0.813
**Estimated glomerular filtration rate (mL/min/1.73 m^2)**	68.0 (45.0; 90.0)	77.2 (58.0; 90.0)	75.0 (55.0; 90.0)	0.300
**Left ventricular ejection fraction (%)**	56.0 (50.0; 60.0)	55.0 (45.0; 60.0)	60.0 (40.0; 60.0)	0.602
**Insulin usage (n, %)**				
**No insulin usage**	32 (41.0%)	29 (41.4%)	39 (59.1%)	0.063
**Basal insulin supported oral therapy**	7 (8.97%)	7 (10.0%)	3 (4.54%)	0.088
**Fixed-ratio combination therapy**	1 (1.28%)	4 (5.71%)	0 (0.00%)	0.079
**Multiple-dose injection therapy**	38 (48.7%)	30 (42.9%)	24 (36.4%)	0.333
**Non-insulin antidiabetic drug usage (n, %)**				
**Metformin**	59 (75.6%)	51 (72.9%)	49 (74.2%)	0.923
**Sulfonylurea**	19 (24.4%)	18 (25.7%)	20 (30.3%)	0.717
**Dipeptidyl peptidase-4 inhibitor**	11 (14.1%)	6 (8.57%)	14 (21.2%)	0.111
**Glucagon-like peptide-1 receptor agonists**	1 (1.28%)	1 (1.43%)	5 (7.56)%	0.097
**Comorbidities (n, %)**				
**Hypertension**	44 (56.4%)	47 (67.1%)	48 (72.7%)	0.113
**Chronic coronary artery disease**	21 (26.9%)	15 (21.4%)	20 (30.3%)	0.651
**Diabetic retinopathy**	14 (17.9%)	11 (15.7%)	15 (22.7%)	0.574
**Peripheric sensory neuropathy**	25 (32.1%)	32 (45.7%)	29 (43.9%)	0.310
**Chronic kidney disease**	31 (39.7%)	21 (30.0%)	33 (50.0%)	0.058
**Active malignancy**	2 (2.56%)	2 (2.86%)	1 (1.51%)	1.000

Supplementary Material S1. Schematic flowchart showing the exclusion criteria used for patient selection.

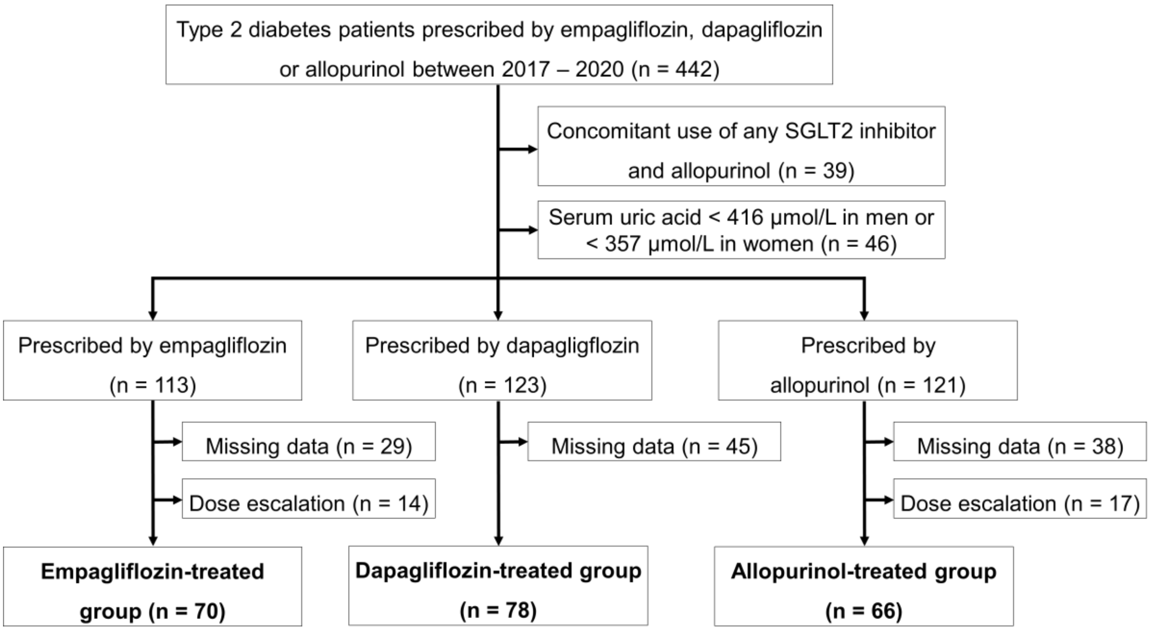

Supplement: Supplementary file 1 [file medsci-14-00421-s001.zip › medsci-4430785-supplementary.pdf]
